# Feasibility and acceptability of systematic screening for depression among people with HIV in Senegal: a qualitative study among various stakeholders

**DOI:** 10.1186/s12888-026-07812-9

**Published:** 2026-01-27

**Authors:** M. Guichard, M. Plazy, H. A. Lam, I. Ndiaye, A. Jaquet, N. F. Ngom, M. Seydi, C. Bernard, the IeDEA West Africa Cohort Collaboration

**Affiliations:** 1 University of Bordeaux, National Institute for Health and Medical Research (INSERM) UMR 1219, Research Institute for Sustainable Development (IRD) EMR 271, Bordeaux Population Health Centre, 146 rue Léo Saignat, Bordeaux Cedex, 33076 France; 2 Laboratory of Sociology, Anthropology and Social Psychology (LASAP), ETOS, Cheikh Anta Diop University, Dakar, Senegal; 3 Psychiatry Department, CHNU de Fann, Dakar, Senegal; 4 Outpatient Treatment Center (CTA), CHNU de Fann, Dakar, Senegal, Bambey, Senegal; 5 Infectious and Tropical Diseases Department, CHNU de Fann, Dakar, Senegal; 6 Department of Medecine at Alioune Diop University, Bambey, Senegal

**Keywords:** Depression, Screening, HIV, West Africa, Senegal

## Abstract

**Background:**

Depression is highly prevalent in people with HIV (PWH) in sub-Saharan Africa and affects their daily lives and the HIV care continuum. However, depression screening is not systematic in HIV services, limiting appropriate care. In the present study, we assessed the feasibility and acceptability of systematic screening for depression among PWH in Senegal.

**Methods:**

Semi-structured individual interviews were conducted from April–July 2023 in Dakar with 18 health professionals (doctors, social workers, community health workers), including some who had experience screening for depression in HIV services, 3 representatives of PWH associations, 2 representatives of the health system, and 5 PWH suffering from depression. The interview guides explored perceptions of depression, as well as facilitators, barriers, and implementation needs at the individual, organizational and structural levels. The Consolidated Framework for Implementation Research (CFIR) conceptual framework was used to structure the study and guide the data analysis. Thematic analysis was conducted via MAXQDA analysis software.

**Results:**

Both patients and healthcare professionals perceived depression screening well. Patients reported positive perceptions related to screening (i.e. relief, opportunity to express themselves). But specific needs were highlighted for its systematic implementation. Healthcare professionals first reported the importance of being trained in mental health. Organizational needs included the definition of a clear depression screening and care pathway adapted to patients (schedules, confidentiality, adapted tools). In addition, systemic barriers to screening were highlighted, especially due to social norms and stigma associated with mental health (i.e., depression is often linked to madness). The interviewed participants thus emphasized the need to improve mental health care literacy to fight stigmatization and the integration of mental health services in HIV care.

**Conclusions:**

Systematic screening for depression appeared acceptable in Senegal, where task delegation is recommended, but its feasibility requires addressing several barriers and considering important needs, including formal integration into HIV services. The study results should help frame innovative and adapted approaches to implement systematic screening of depression through HIV services in sub-Saharan Africa.

**Clinical trial number:**

Not applicable.

**Supplementary Information:**

The online version contains supplementary material available at 10.1186/s12888-026-07812-9.

## Background

Depression affects approximately 280 million people worldwide, representing 5% of the world’s adult population [1, 2], and its prevalence is two to three times higher in people with HIV (PWH) than in the general population [3]. In fact, PWH are particularly vulnerable to depression [4] due to inflammatory processes [4], the stigma and discrimination associated with HIV/AIDS, and the fear of premature death [5].

In sub-Saharan Africa (SSA), a previous literature review estimated that 9 to 32% of PWH had moderate to severe depressive symptoms [6]. Depression can impact on the continuum of HIV care, being associated with poorer adherence to antiretroviral treatment (ART) [7] and adversely affecting clinical HIV outcomes such as lower CD4 T-cell recovery [8], faster progression to AIDS and increased mortality [4].

Unfortunately, although several treatments for depression are effective and accepted by PWH in SSA [9–11], depression screening is not routinely available in many HIV care services. This gap prevents the optimal management of depression and could threaten the sustainability of depression treatment interventions. The implementation of systematic depression screening (i.e. screening of all PWH attending to their routine visit whether or not they have depressive symptoms) could be an effective strategy for depression management compared to selective screening [12], and should therefore be a priority in HIV services [4]. However, more research is needed to address knowledge gaps and facilitate the systematic integration of mental health services into HIV care services in low- and middle-income countries [13, 14], particularly in West Africa, where no studies have been conducted on this topic.

In Senegal, a limited resource francophone West African country with over 16 million inhabitants as of 2020 [15], approximately 44,000 people lived with HIV in 2023 (https://aidsinfo.unaids.org/). Data on the prevalence of depression in PWH in Senegal are scarce but one study reported a prevalence of 18% [16]. Another study reported a similar prevalence specifically in PWH aged 50 years and older [17]. A large mental health care gap is however observed: only 38 psychiatrists provided care in 2018 according to the Ministry of Health and Social Action [18]. While screening for depression is not available in Senegalese HIV settings, there is a need to improve mental health care access for PWH. In light with this, the National Strategic Plan of the Senegalese Mental Health Division 2024–2028 highlighted the importance to develop partnerships with other national programs, such as the HIV/AIDS program, to integrate mental health services in HIV care [15]. In this context, we aimed to assess the acceptability and feasibility of implementing systematic depression screening in HIV care services in Senegal by triangulating the perspectives of different stakeholders.

## Methods

### Study setting

The study took place in Dakar, the capital of Senegal, located in the west of the country. Dakar hosts many of Senegal’s medical and research facilities, making them less accessible to patients from outside the region [19]. The HIV prevalence in Dakar (0.4%) is similar to the national prevalence (0.3%) [20]. In this context, the “Intervention depression” project (March 2019 - June 2024) [9] aimed to assess the acceptability and feasibility of group Interpersonal Therapy (IPT) to treat depression in four different HIV care services in Dakar: (i) the Infectious and Tropical Diseases Unit (SMIT) and (ii) the Outpatient Treatment Center (CTA) at the Fann National University Hospital Center (level 3 hospital); (iii) the Youssou Mbargane Diop Hospital in Rufisque (level 1 hospital); and (iv) the Hyacinthe Thiandoum Cardinal Health Promotion Center (care center) in the suburbs of the capital. It was conducted within the West Africa network of the International Epidemiological Databases to Evaluate AIDS (IeDEA West Africa) of the U.S. National Institutes of Health (https://www.iedea.org/regions/west-africa/).

During the “Intervention depression” project, patients were screened for depression during their routine HIV clinical visits. Screening was conducted by physicians at SMIT and CTA and by social or community health workers at the other two sites. The screening tool used was the Patient Health Questionnaire-9-items (PHQ-9) [21], a widely used screening tool based on *Diagnostic and Statistical Manual for Mental Disorders* (DSM-IV) key depressive symptoms [21]. Other physicians and social workers/community health workers involved in the project were responsible for confirming the diagnosis of depression and providing treatment, respectively. PWH with positive screening were systematically referred to the physician in charge for confirmation of a depression diagnosis. If the diagnosis was confirmed, PWH was referred to a social worker or a community health worker trained in group IPT. As defined in the WHO manual [22], group IPT includes an individual session and 8 group sessions, one per week. Suicide risk was systematically assessed for each patient who reported anything other than “not at all” for the last item of the PHQ-9 (i.e. “In the last two weeks, have you thought about hurting yourself in any way?“). We used the Columbia Suicide-Severity rating scale, a standardized tool to assess the level of risk (passive, active-low, active-moderate, active-severe) [23]. Patients with an active moderate-to-severe risk were referred to the psychiatrist who determined their eligibility for participation in group sessions. In cases where participation was deemed unsuitable, the attending psychiatrist would propose an alternative course of treatment.

### Study design and population

This qualitative study is a sub-study of the “Intervention depression” project, subsequently motivated by observed challenges reaching patients and discrepancies in screening practices for depressive disorders. The study involved various stakeholders to obtain a comprehensive understanding of the feasibility and acceptability of systematic depression screening. The participants included: (i) healthcare professionals (physicians, social workers, community health workers) with and without prior experience in depression screening, (ii) representatives of the health care system (National HIV/AIDS Program and Mental Health Division (MHD) of the Ministry of Health), (iii) representatives of PWH associations, and (iv) PWH suffering from depression and who were participating in the “Intervention depression” project (excluding individuals not fluent in French). The participants were purposively selected to ensure heterogeneity in their profiles and experiences across stakeholder groups [24]. The selection criteria included age, gender and healthcare centers for healthcare professionals; and age, gender and time since depression screening for PWH. Representatives of the health system and of the PWH associations were identified and contacted by the study’s principal investigator. The present study adhered to the Standards for Reporting Qualitative Research Guidelines [25].

### Conceptual framework

In the field of implementation research, Proctor et al. (2011) defined acceptability as the “*perception among implementation stakeholders that a given treatment*,* service*,* practice*,* or innovation is agreeable*,* palatable*,* or satisfactory*”. They further defined feasibility as “*the extent to which a new treatment*,* or innovation*,* can be successfully used or carried out within a given agency or setting*” [26]. In this study, we aimed to understand the barriers, facilitators, and needs related to the implementation of depression screening on the basis of the perceptions and experiences of various stakeholders. To this end, our research was structured using the first version of the Consolidated Framework for Implementation Research (CFIR), published in 2009. The CFIR was developed to guide the systematic evaluation of multilevel implementation contexts and to identify factors that may influence intervention implementation and effectiveness [27]. The CFIR comprises five domains: (i) the intervention, (ii) the inner setting, (iii) the outer setting, (iv) the individuals involved and (v) the process by which the implementation of the intervention is achieved. For each of these five domains, the CFIR provides a list of explicitly defined constructs for which data can be collected. In the present study, the CFIR was specifically employed to develop the data collection tools (interview guides) and to guide the data analysis.

### Data collection

Semi-structured face‒to-face interviews were conducted in French from May to July 2023 by the first author (MG), a French research intern enrolled in a master’s program in global health and trained in qualitative research, who worked under the supervision of senior French researchers (CB, MP) and a Senegalese social anthropologist (HAL). All the interviews were conducted in private rooms at one of the four project centers, with the exception of those with health system representatives, conducted at their respective workplaces. The interviews were conducted one-on-one, with the exception of one interview involving two PWH, upon their request.

The interview guides (see Additional file 1) were developed by selecting the most relevant CFIR domains for this study and by linking the associated constructs to the implementation of depression screening (see Additional file 2). The guides were adapted to each stakeholder and explored perceptions and knowledge about depression, attitudes toward depression screening, experiences in mental health care and implementation criteria (for those who experienced depression screening).

The interviews were recorded with a dictaphone only after the participants had given their consent. For one patient who refused to be recorded, detailed notes were taken instead. The duration of the interviews ranged from 10 to 40 min for PWH and from 30 to 70 min for other stakeholders. The first author then transcribed all audio-recorded interviews. Data collection continued until theme saturation was reached (i.e. until no new themes emerged).

### Data analyses

The first author (MG) analysed transcriptions using a classical thematic analysis method [24, 28]. This analysis was guided by a coding tree (see Additional file 3) which was developed from the conceptual framework, including (i) different constructs of the CFIR adapted to the research context and (ii) whether the constructs represented barriers, facilitators, or needs related to the implementation of depression screening in HIV care services. The coding tree was developed by the first author (MG) in collaboration with the second author (MP, a French senior lecturer in Public Health, specialised in implementation research) and the last author (CB, a French researcher, scientific coordinator of the “Intervention Depression” project). To ensure the reliability of the results: 1/ Data were triangulated by validating the results with information collected during informal conversations with key individuals; 2/ All findings were discussed and validated with Senegalese colleagues, including a social anthropologist (HAL), clinicians working in HIV services (NFG, MS) and a local psychiatrist (IN); 3/ Excerpts from participants’ interviews were included in the results, allowing for validation of the interpretations. The coding process was performed using the MAXQDA software, version 2022.7 [29].

## Results

### Participants’ characteristics

A total of 28 individuals were interviewed. The participants’ characteristics are detailed in Table 1. Among the 18 healthcare professionals (55.6% female, median age: 49.5 years), eight were physicians, six were social workers, and four were community health workers. More than half of the health care professional had participated in the “intervention depression” project: they were made aware of the importance of mental health and depression and were involved in the management of depression, but were not necessarily responsible for screening. Six had previous experience with depression screening (as part of the “Intervention depression” project). Among the five PWH interviewed, three were female, and the median age was 45 years. In addition to health care professionals and patients, three representatives of PWH associations and two representatives of the healthcare system were included.

**Table 1:** Participants’ characteristics

Stakeholders	*N*	Median age	Women/Men (*n*)	Involved in the “Intervention depression” project (*n*)	Depression screening experience (*n*)
Healthcare professionals	18				
Physicians	8	46,0	4/4	5	3
Social workers	6	47,5	4/2	4	2
Community health workers	4	48,5	2/2	3	1
Representatives of PWH associations	3	50,0	2/1	-	-
Representatives of the healthcare system	2	47,0	0/2	-	-
PWH	5	45,0	3/1	5	5

The participants were mostly favorable regarding depression screening, but they also highlighted several barriers and needs for depression screening to be systematically implemented in HIV services.

### FACILITATORS for depression screening

#### Relevance of including systematic depression screening within the comprehensive care offered to PWH

All the interviewees agreed that depression screening in PWH is a necessity for PWH, as mental disorders are relatively common in this population, especially in some vulnerable populations, such as key populations, newly diagnosed individuals, adolescents, and elderly individuals. In this context, healthcare professionals explained the importance of addressing both physical/somatic and mental health in patient care. They suggested that depression screening, if systematic, could be a critical step in identifying and then treating patients who might not be suspected of having depression, as well as leading to better HIV-related outcomes and improved overall health and well-being.

*Appearances are often deceptive*,* and only screening can tell us if the person is going through this stage or not. So I find it important and crucial to do it.* (Social worker, 58 years)

*Screening is beneficial because it is preventive. Because when you screen*,* that’s what screening is all about. Because it means that you are perhaps getting ahead of the problem*,* instead of waiting for obvious signs to appear.* (Representant of the system)

*You know*,* depression is connected to everything. Therefore*,* if we can detect it early*,* we can prevent certain things. Because an HIV-positive person who becomes depressed can affect their social life*,* their professional life and even their medical follow-up.* (Physician, 57 years old)

*Identifying it [depression] may enable them to receive better care and have a better response*,* which will impact the outcome of the program and the response. (Social worker*,* screening*,* 45 years old).*

PWH also reported that depression screening was a form of relief and an opportunity to express themselves. They highlighted the need of a global care.

*We were not always in the mood*,* so we need help on all fronts (…) Yes*,* I was relieved when I answered the questionnaire with the doctor. Because he let me know it was confidential*,* and he guided me*,* which made me feel comfortable sharing what’s in my heart.* (PWH, 26 years old)

*She arrived and asked me all [the PHQ-9 questions]. She took me out of my body. (PLHIV, 45 years old)*

*We weren’t always feeling well*,* so you need help in every way. (PLHIV, 26 years old)*

### BARRIERS for depression screening

#### Depression faced with cultural beliefs, social norms and stigmatization

Among the healthcare professionals and association representatives who were interviewed, 13 described depression as synonymous with madness or associated with “*being crazy*” in Senegalese culture. They also reported that in Senegal, mental disorders, including depression, are often associated with spirits or demons (also called “djinns” and “raps”, respectively, in Wolof, the national language of Senegal). Those who had already conducted depression screening added that these cultural beliefs could lead to a certain denial of their condition among patients, thus preventing them from accepting depression screening.

*[For patients] It is like you’re crazy. It is from experience*,* you know*,* if I say depression*,* I know that after the answers will be ‘yes*,* I’m fine*,* everything’s good*,* I’m very well’.* (Physician, 49 years)

*Here*,* mental illness is much more associated with stories of spirits*, etc.,* so when a person is affected by it*,* it is said that either the parents have sinned*,* that they did not make the sacrifices they were supposed to*,* or an ancestor has offended a djinn*,* or a parent has bewitched them*,* or a colleague*,* a neighbor.* (Social worker, 50 years)

Another cultural barrier to depression screening reported by both patients and healthcare professionals was that it can be considered inappropriate to share one’s problems and admit that things are not going well, especially among men. The difficulty in opening up is also due to cultural behavior related to the idea that others can suffer more than you.

*As for men*,* they do not accept at all that they are depressed in front of a woman. Perhaps it is related to our culture*,* that a man must always remain strong; that is what we have been taught.* (Social worker, 45 years old)

*I think it is part of us. Generally*,* we often do not share our problems. We say if you have problems*,* there are many others who have them*,* and you should not lament about your issues. That is why everyone keeps their problems.* (PWH, 19 years old)

*You should not complain about your situation because there’s always someone worse off than you.* (PWH, 26 years old)

According to healthcare professionals, cultural beliefs and social norms associated with depression, as well as the challenges of opening up, can lead to stigmatization of affected individuals and act as a social barrier to depression screening.

*Depression is another pathology*,* like HIV*,* and it is negatively perceived by society*,* so there is a negative connotation. The fear of being stigmatized can already be a hindrance to screening and especially accepting one’s condition.* (Physician, 51 years old)

#### Lack of knowledge about depression

In addition to the cultural beliefs and social norms, understanding of depression seemed to be limited among PWH, who often see it as “*stress*”. Similarly, a representative of a PWH association reported that depression could be perceived as a symptom of HIV, and that PWH may have difficulties to distinguish between these two conditions.

*She [the social worker] was trying to explain me that with the illness*,* there are depressions*,* but they are not depressions that can be classified as mental disorders. Depressions*,* due to the illness [HIV]*,* you experience things*,* you endure things*,* and you have no one to talk to about it.* (PWH, 50 years old)

*Screening for depression in PWH is relevant. So that at least people can know how to differentiate things*,* to say that this is depression*,* you understand (…); that is*,* they attribute everything to HIV.* (Representative of a PWH association, 62 years old)

Physicians, whether involved in the “Intervention depression” project or not, generally had some understanding of mental health and depression, although they explained that many symptoms overlap with those of HIV (such as loss of appetite, lack of sleep, etc.), so that patients or other professionals do not realize that they could be related to another condition, such as depression.

*I felt that some [professionals] did not understand what depression was as such*,* especially in this context of HIV. Because the classic signs of depression may not be present. In addition*,* you have to look for other signs that*,* if they are present*,* might indicate depression.* (Physician, 43 years old)

#### Time needed for depression screening

Some healthcare professionals reported that screening for depression was a time-consuming task in addition to their regular consultations with PWH, and therefore doubted the feasibility to implement it systematically. Healthcare professionals also explained that the time required to administer the screening tool was unpredictable and varied from one patient to another, depending on their understanding of the questions.

*Well*,* logically*,* if there are many patients*,* it is clearly complicated. Because it takes time. For example*,* when I was involved in that project*,* I conducted the screening and had consultations with patients. Therefore*,* it takes more time*,* and it may not be done correctly or systematically.* (Physician, 49 years old)

Time required for depression screening could also be a barrier for some patients, as they might lack of time when visiting the healthcare center.

*Sometimes the patient lives far away*,* and they do not have much time to wait for us to do the screening.* (Community health worker, 48 years old)

In health centers where screening was carried out by the social team, this difficulty was exacerbated when the schedules of the social and medical services differed.

*Unfortunately*,* they [patients] think that to be seen early enough*,* they should be there [at the health center] at 6 am/7 am*,* even though the consultation starts at 8 am. Therefore*,* they think they will go directly to the consultation office*,* wait there*,* sign up on the list*,* and wait. Unfortunately*,* the social service’s activity [where depression screening was done] starts at 8 am/9 am.* (Physician, 33 years old)

### NEEDS for implementing systematic screening

In order to address these different barriers, the participants highlighted several needs that should be considered for systematic depression screening.

#### Increase awareness in both health centers and the community

Most healthcare professionals repeatedly emphasized the need for all healthcare professionals to receive regular training to be able to screen for depression, particularly in order to cope with staff turnover in healthcare facilities.

*You always need to be trained*,* especially for mental health. I think it is not just anyone who can handle it [depression screening].* (Physician, 51 years old)

*You need to train people in this field because (…) you know that there are people [healthcare workers] who come and go [to/from healthcare centers]. They are not static. So it would be good from time to time to update*,* recycle people in this field.* (Social worker, 45 years old)

In addition, interviewees highlighted the importance of raising community awareness of depression, by demystifying the illness, in order to encourage people to seek care at healthcare facilities and thus facilitate the detection of depression.

*First*,* you need to have a trained core; the big leaders*,* I mean the presidents of associations and others. They need to know for themselves what mental health is.* (Representative of the PWH association, 48 years old)

#### Available and trustworthy staff

The interviewees questioned the need to identify who should screen for depression in healthcare centers. Having time to do screening is essential to avoid rushing appointments and the risk of missing certain signs of depression. In this context, while doctors often have a heavy workload, several healthcare professionals agreed that the social team could be well-placed to manage depression screening, especially as psychosocial care is part of their role.

*In the team*,* everyone can conduct the screening. However*,* ideally*,* it is best done with the social team (…)*,* it is primarily their job to provide psychosocial support*,* and they have much more time to dedicate to it.* (Social worker, 45 years old)

*Well*,* if we consider the workload here*,* currently*,* given the current situation*,* it is the social workers [who could undertake depression screening]. Because as I see the consultation unfolding*,* if a physician who has to administer the questionnaire*,* they will have to discuss the logistics*,* as I mentioned*,* what time of the day*,* what day of the week. (…) Yes*,* a doctor can do that*,* but there truly needs to be an organization behind it.* (Physician, 57 years old)

In addition, both patients and healthcare professionals stressed the importance of depression screening carried out by professionals whom patients consider to be “trustworthy”. According to physicians and social workers with experience of depression screening, knowing patients could facilitate the process (i.e. screening).

*First*,* it is important to make the person feel comfortable and build trust.* (PWH, 26 years old)

#### Definition of a screening circuit

To implement systematic depression screening in HIV care settings, healthcare professionals have expressed the need to define a clear care pathway, with an unidentifiable location to preserve confidentiality and limit stigmatization.

*The problem here is the location of the social workers’ office. If we have to set up a pathway where patients have to visit*,* it is a confidentiality issue that will arise. (…) In this room*,* we do ultrasounds*,* and the people sitting there cannot know who came for an HIV consultation*,* who came for an ultrasound (…). For me*,* if we still have to do it [depression screening]*,* we need to identify another place that would be much more confidential to be able to do it.* (Physician, 57 years old)

In health centers where depression screening was provided by social workers, health professionals also indicated that having an office close to the pharmacy could facilitate systematic screening and avoid missing patients.

*Well*,* I would say trying to reach everyone*,* touch all the people who come by*,* maybe see the entrance to the pharmacy. I was side by side with the pharmacy at the time; I see all the patients who come as well.* (Social worker, 58 years old)

In this context, one of the representatives of the healthcare system emphasized the need for collaboration between the different healthcare professionals within the HIV service. In particular, it seems that the care pathway needs to be clearly defined according to each care setting so that the patient knows where to go, and healthcare professionals are aware of the patient’s pathway, thus facilitating communication between them when necessary for the patient’s care.

*Sometimes*,* there are these different units [in the HIV service] that do not communicate. Only the documents circulate (…) This is sometimes what is missing this dialog because the patient should be at the center [of the discussion].* (Representative of Ministry)

#### Adaptation of a screening tool

Healthcare professionals who used the depression screening tool in the “Intervention Depression” project (i.e., the PHQ-9) described it, as relatively easy and clear to use. They reported that most of the PHQ-9 questions were relevant, with some patients answering the questions before they were asked. However, they explained that two questions did not seem to be adapted to Senegalese realities: item 9, which refers to suicide, a forbidden act in the Muslim religion that is widespread in Senegal, and item 8 (i.e., “Moving or speaking so slowly that other people could have noticed, etc.”), which was not always well understood by some patients.

*Where we mentioned suicide*,* maybe*,* you know*,* the Muslim religion prohibits suicide*,* so quite a few people tell you that truly*,* my religion does not allow it.* (Social worker, 56 years old)

Professionals also emphasized the need to have a tool available in all local languages.

*For example*,* in the tool*,* everything is written in French*,* […]. Screening can sometimes be challenging. We can ask the question; we can explain each word in Wolof*,* but sometimes we have inconsistencies in their response.* (Community health worker, 48 years old)

#### Integration of mental health into HIV care services

The interviewees underlined the need to integrate depression screening into all HIV care services at a national level, highlighting the important role of decision-makers. More specifically, they suggested that the screening tool should be included in data collection tools, as is the case for other comorbidities such as tuberculosis.

*Therefore*,* what I recommend if we have to expand is to include it in the service’s package of activities. […] You know*,* if it has to come separately*,* it [depression screening] might not be taken into account*,* might not be embraced by the service.* (Social worker, 45 years old)

*Therefore*,* the integration of systematic screening into data collection tools will help us. With that*,* we can easily integrate it. Like what is done*,* for example*,* with tuberculosis*,* you are given a form*,* and any provider can perform screening.* (Physician, 51 years old)

More broadly, several health professionals have expressed a willingness to extend and integrate screening for depression systematically in practice for people suffering from other chronic conditions, such as hepatitis, hypertension and diabetes, as these conditions are known to affect patients’ mental health.

*Individuals receiving treatment for chronic illnesses often experience depression. Because when we say chronic illnesses*,* we mean lifelong illnesses*,* you have to take medication every day*,* some people have to go on diets…* (Physician, 33 years old)

Furthermore, to integrate depression care and thus systematize screening in healthcare centers, several participants (healthcare professionals, representatives of PWH associations and of the Ministry of Health) emphasized the importance of being able to offer treatment and follow-up after screening. This idea goes hand in hand with the need to have the necessary skills to treat patients after screening.

*We can do the screening for depression*,* no problem. However*,* what will the patient gain if there is no follow-up? […] if follow-up is not there*,* does the screening have its reason for being?* (Community health worker, 50 years old)

## Discussion

**Fig. 1:**
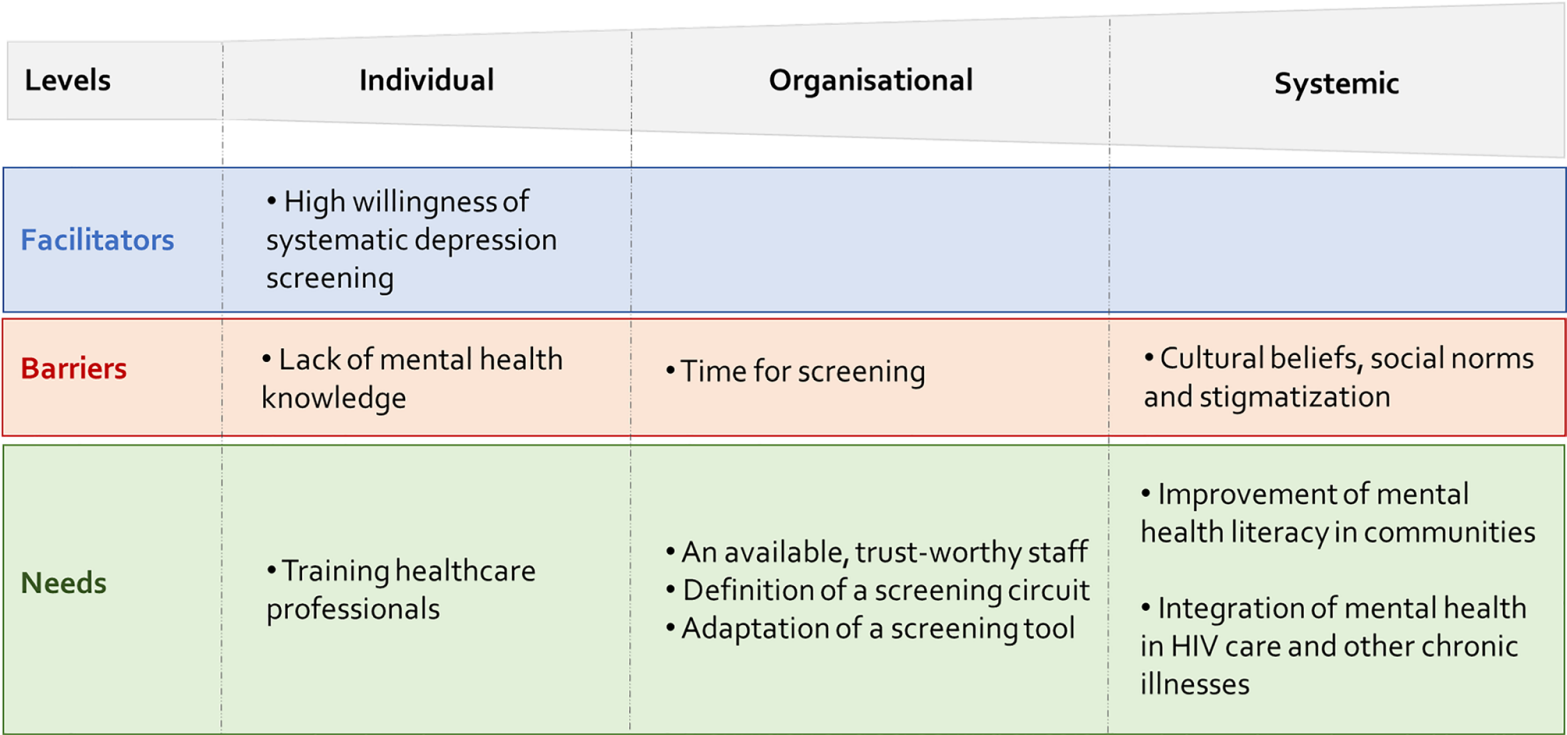
Sum-up of facilitators, barriers and needs for integrating routine depression screening into HIV care centres in Senegal

Our study identified several facilitators, barriers, and needs related to the implementation of systematic screening for depression in HIV care centers in Senegal. These factors can be summarized at three levels: individual (patients and healthcare professionals), organizational, and systemic (Fig. 1).

At the individual level, all interviewees recognised the importance of and need for systematic depression screening in HIV services, as it has also been reported in Malawi [30]. The main barrier was a lack of understanding of depression, which was often perceived more as stress or a symptom of HIV disease, especially among PWH. A study conducted in Botswana showed that PWH were not able differentiate depression from sadness [31]. Depression also appeared to be difficult for healthcare professionals to diagnose as certain symptoms overlap with those of HIV (loss of appetite, sleep disturbances). Medical knowledge about this condition appears to be inconsistent, as previous studies in Botswana, Colombia and Malawi have shown [31–33]. This highlights the need for adequate training in depression screening for healthcare professionals as well as the development of clear guidelines, especially to cope with staff turnover in services and to ensure its systematic use. Ongoing supervision and support have also been shown to be necessary to ensure effective depression screening in Cameroon and Malawi [14, 34].

At the organizational level, the main barrier identified was the time required for depression screening, which was perceived as an additional and time-consuming task, particularly for physicians and some patients, as also reported in several previous studies in different contexts [31, 32, 35, 36]. Consequently, the need for available and trustworthy staff to not rush the questionnaire administration and to listen to the patient has been reported. Indeed, a study conducted in Malawi showed that PWH were more likely to disclose their suicidal ideation if they did not feel rushed during the appointment [34]. In this context, social workers and community health workers appear to be well suited to conduct depression screening, given that psychosocial support and counseling are part of their activities. In line with other studies conducted in sub-Saharan Africa, depression screening should be added to the HIV counseling and screening sessions, which are currently only partially used as an opportunity to identify and initiate treatment for mental health problems [36, 37]. In addressing the question of “where and how to screen”, workflows could be adjusted by integrating depression screening into existing routines, such as ART refill visits, HIV counseling sessions, or pharmacists’ visits, to minimize additional time burden and avoid separate appointments. Adapting the care pathway is critical to ensure that depression screening for PWH is conducted to ensure confidentiality, especially due to HIV-related stigmatization [31]. The screening sites need to be private and quiet [30]. Increased collaboration between healthcare professionals involved in HIV care was reported as another need. For example, pharmacists could serve as referral points for supplementary support and educational tools that might benefit patients [38]. Finally, another important organizational aspect is the need to have a tool adapted to the local culture and available in local languages to facilitate its administration, improve comprehension, and ultimately saving staff time, as previously documented [30, 39, 40]. A wolof version of the PHQ-9 is now available in Senegal.

At the systemic level, cultural beliefs and stigmatisation surrounding depression were identified as significant barriers, as reported in other sub-Saharan countries [14, 31, 37]. inconsistent with the findings of our study, depression was also perceived as a form of madness in Botswana [31] and as being caused by witchcraft in Uganda [36]. In Ghana, pregnant or postpartum women feared being labelled “not in their right mind” if they were diagnosed with a mental disorder [35]. The importance of enhancing mental health literacy within communities, particularly through community leaders, was emphasized to increase awareness of depression and reduce stigmatization. Finally, the interviewees stressed the need to integrate mental health into HIV care, including both treatment and follow-up after depression screening. Indeed, the absence of treatment options, whether pharmacological or therapeutic, represents a significant barrier to the effective management of mental disorders [31, 41]. In this context, while a lack of clear policy frameworks and programs often hinders adequate training and resource allocation for mental health services; it is essential that clear management policies are addressed in each healthcare system [42]. In this context, the Senegalese Ministry of health should: 1/integrate systematic screening into HIV care guidelines; 2/ adopt a specific screening tool and incorporate it in HIV data collection system; 3/ train staff on mental health and depression and organize refresher trainings; 4/ reinforce community awareness campaign on mental health and depression to decrease stigma and increase help-seeking;

This study is the first to explore the feasibility and acceptability of systematic depression screening among PWH in West Africa. The inclusion of different stakeholders, including both healthcare professionals and beneficiaries, allowed for the exploration of perceptions and experiences, providing a holistic perspective. The use of the CFIR conceptual framework throughout the study’s various phases facilitated the identification of multilevel determinants influencing the implementation of systematic depression screening. Additionally, while data collection and initial analysis were conducted by French researchers, the results were discussed and validated with Senegalese colleagues, including a socio anthropologist, several clinicians and a psychiatrist, thus ensuring cultural and contextual relevance in the interpretation of the results. However, the study had several limitations. Saturation was probably not reached, particularly for PWH. The small number of PWH included (*n* = 5) was mainly due to accessibility challenges related to the political context in Senegal from late May to early June 2023. Also, since the interviews were conducted by a French intern as part of her Master of Public Health program, it was not possible to include patients who were not fluent in French, which may have led to some selection bias. In addition, it was sometimes difficult to use the term “depression” in certain interviews, a term stigmatized in Senegal, requiring a case-by-case review of how the questions were asked. Finally, for ethical reasons, we did not explore the perspectives of PWH who were not included in the “Intervention Depression” project, which limited insights into broader aspects of depression screening implementation.

## Conclusions

Systematic depression screening in HIV care centers appeared acceptable to various stakeholders in Senegal, but its feasibility requires addressing several barriers and considering important needs, including the formal integration of mental health services into HIV care centers with the elaboration of specific pathways depending on the characteristics of the setting. In order to fight stigmatization associated with depression, it will also be important to raise awareness on mental health at community level.

## Supplementary Information

Below is the link to the electronic supplementary material.

Supplementary Material 1

Supplementary Material 2

Supplementary Material 3

## Data Availability

The datasets presented in this article are not readily available because they contain confidential information that could compromise participant privacy but can be made available with nonidentifiable aspects from the corresponding author upon reasonable request. Requests to access the datasets should be directed to [charlotte.bernard@u-bordeaux.fr].

## References

[1] Santomauro DF, Herrera AMM, Shadid J, Zheng P, Ashbaugh C, Pigott DM, et al. Global prevalence and burden of depressive and anxiety disorders in 204 countries and territories in 2020 due to the COVID-19 pandemic. Lancet. 2021;398(10312):1700–12. 34634250 10.1016/S0140-6736(21)02143-7 PMC8500697

[2] World mental health report [Internet]. [cited 2023 Jan 16]. Available from: https://www.who.int/teams/mental-health-and-substance-use/world-mental-health-report.

[3] Impact of psychiatric conditions on health-related quality of life in persons with HIV infection. | American Journal of Psychiatry [Internet]. [cited 2023 May 9]. Available from: https://psychiatryonline.org/doi/full/10.1176/appi.ajp.157.2.248. 10.1176/appi.ajp.157.2.248 10671395

[4] Abas M, Ali GC, Nakimuli-Mpungu E, Chibanda D. Depression in people living with HIV in sub-Saharan Africa: time to act. Trop Med Int Health. 2014;19(12):1392–6. 25319189 10.1111/tmi.12382

[5] Akena DH, Musisi S, Kinyanda E. A comparison of the clinical features of depression in HIV-positive and HIV-negative patients in Uganda. Afr J Psychiatry. 2010;13(1):43–51. 10.4314/ajpsy.v13i1.53429 20428598

[6] Bernard C, Dabis F, de Rekeneire N. Prevalence and factors associated with depression in people living with HIV in sub-Saharan Africa: a systematic review and meta-analysis. PLoS ONE. 2017;12(8):e0181960. 28783739 10.1371/journal.pone.0181960 PMC5544236

[7] The effect of depressive symptoms. and CD4 count on adherence to highly active antiretroviral therapy in sub-Saharan Africa - Peter Memiah, Constance Shumba, Martine Etienne-Mesubi, Solomon Agbor, Mian B. Hossain, Patience Komba, Mercy Niyang, Sibhatu Biadgilign, 2014 [Internet]. [cited 2023 May 9]. Available from: 10.1177/2325957413503368. 10.1177/2325957413503368 24114726

[8] Depression symptoms, social support and overall health among HIV-positive individuals in Kenya - Caroline Kingori, Zelalem T Haile, Peter Ngatia, 2015 [Internet]. [cited 2023 May 9]. Available from: https://journals.sagepub.com/doi/10.1177/0956462414531933 10.1177/0956462414531933 24759561

[9] Bernard C, Font H, Ziadeh S, Tine JM, Diaw A, Ndiaye I, et al. Management of depression in people living with HIV/AIDS in Senegal: Acceptability, feasibility and benefits of group interpersonal therapy. Camb Prisms Glob Ment Health. 2023;10:e36. 10.1017/gmh.2023.31 PMC10579691 37854409

[10] Bolton P, Bass J, Neugebauer R, Verdeli H, Clougherty KF, Wickramaratne P, et al. Group interpersonal psychotherapy for depression in rural UgandaA randomized controlled trial. JAMA. 2003;289(23):3117–24. 12813117 10.1001/jama.289.23.3117

[11] Asrat B, Lund C, Ambaw F, Schneider M. Acceptability and feasibility of peer-administered group interpersonal therapy for depression for people living with HIV/AIDS—a pilot study in Northwest Ethiopia. Pilot Feasibility Stud. 2021;7:147. 34321104 10.1186/s40814-021-00889-x PMC8317371

[12] Okimat P, Akena D, Opio D, Mutabazi T, Sendaula E, Semitala FC, et al. Screening PLHIV for depression using phqs: A RCT comparing non-selective with selective screening strategy within a primary health care facility in Uganda. PLoS ONE. 2022;17(6):e0270175. 35767586 10.1371/journal.pone.0270175 PMC9242435

[13] Integration of mental. health and HIV interventions — Key considerations.

[14] Grimes KEL, Ebasone PV, Dzudie A, Nash D, Wainberg ML, Pence BW, et al. Factors influencing integration of mental health screening and treatment at HIV clinic settings in cameroon: a qualitative study of health providers’ perspectives. BMC Health Serv Res. 2024;24(1):519. 38658992 10.1186/s12913-024-10775-w PMC11044447

[15] Plan Stratégique National de la Division Santé Mentale. 2024–2028. https://www.sante.gouv.sn/sites/default/files/Plan%20strate%CC%81gique%20Sante%CC%81%20Mentale%20Senegal%202024-2028%20V.pdf

[16] Poupard M, Ngom Gueye NF, Thiam D, Ndiaye B, Girard PM, Delaporte E, et al. Quality of life and depression among HIV-infected patients receiving efavirenz- or protease inhibitor-based therapy in Senegal. HIV Med. 2007;8(2):92–5. 17352765 10.1111/j.1468-1293.2007.00435.x

[17] Bernard C, Font H, Diallo Z, Ahonon R, Tine JM, N’guessan Abouo F, et al. Prevalence and factors associated with severe depressive symptoms in older West African people living with HIV. BMC Psychiatry. 2020;20(1):442. 32912173 10.1186/s12888-020-02837-0 PMC7481548

[18] Petit V. Mental health: an underestimated development issue, in Y. Charbit, editor, Population ans development issues. [Internet]. ISTE-WILEY. 2022. pp. 157–181. Available from: https://www.iste.co.uk/book.php?id=1877.

[19] https://www.usaid.gov/sites/default/files/documents/1860/20180213_Senegal_CVA_Report_External.pdf.

[20] Centre National de Lutte contre le SIDA du Sénégal. https://www.cnls-senegal.org/documents/plan-strategique-national-pour-une-riposte-multisectorielle-integree-contre-le-sida-la-tuberculose-les-hepatites-virales-et-les-ist-2023-2030/.

[21] Kroenke K, Spitzer RL, Williams JBW. The PHQ-9. J Gen Intern Med. 2001;16(9):606–13. 11556941 10.1046/j.1525-1497.2001.016009606.x PMC1495268

[22] WHO. Group Interpersonal Therapy (IPT) for Depression. 2016. https://www.who.int/publications/i/item/WHO-MSD-MER-16.4.

[23] Posner K, Brown GK, Stanley B, Brent DA, Yershova KV, Oquendo MA, et al. The Columbia-Suicide severity rating scale: initial validity and internal consistency findings from three multisite studies with adolescents and adults. Am J Psychiatry. 2011;168(12):1266–77. 22193671 10.1176/appi.ajp.2011.10111704 PMC3893686

[24] Miles M, Huberman A, Saldana J. Qualitative data analysis: a methods sourcebook (4th edition). Thousand Oaks, United States of America, SAGE Publications. 2018.

[25] O’Brien BC, Harris IB, Beckman TJ, Reed DA, Cook DA. Standards for reporting qualitative research: a synthesis of recommendations. Acad Med J Assoc Am Med Coll. 2014;89(9):1245–51. 10.1097/ACM.0000000000000388 24979285

[26] Proctor E, Silmere H, Raghavan R, Hovmand P, Aarons G, Bunger A, et al. Outcomes for implementation research: conceptual Distinctions, measurement Challenges, and research agenda. Adm Policy Ment Health Ment Health Serv Res. 2011;38(2):65–76. 10.1007/s10488-010-0319-7 PMC3068522 20957426

[27] Damschroder LJ, Aron DC, Keith RE, Kirsh SR, Alexander JA, Lowery JC. Fostering implementation of health services research findings into practice: a consolidated framework for advancing implementation science. Implement Sci IS. 2009;4:50. 19664226 10.1186/1748-5908-4-50 PMC2736161

[28] Green J, Thorogood N. Qualitative methods for health research. London: SAGE; 2004. p. 262. (Introducing qualitative methods).

[29] Software VERBI. MAXQDA 2024 [computer program]. Berlin (DE): VERBI Software; 2022. Available from: https://www.maxqda.com

[30] Kip EC, Udedi M, Kulisewa K, Go VF, Gaynes BN. Barriers and facilitators to implementing the HEADSS psychosocial screening tool for adolescents living with HIV/AIDS in teen club program in malawi: health care providers perspectives. Int J Ment Health Syst. 2022;16:8. 35101066 10.1186/s13033-022-00520-3 PMC8805413

[31] Molebatsi K, Ho-Foster A, Ntsayagae E, Bikimane B, Bauer AM, Suleiman K, et al. Implementation planning for integrating depression screening in diabetes mellitus and HIV clinics in Botswana. Glob Implement Res Appl. 2022;2(4):384–93. 36340843 10.1007/s43477-022-00062-3 PMC9628413

[32] Bartels SM, Cardenas P, Uribe-Restrepo JM, Cubillos L, Torrey WC, Castro SM, et al. Barriers and facilitators to the diagnosis and treatment of depression in primary care in colombia: perspectives of providers, healthcare administrators, patients and community representatives. Rev Colomb Psiquiatr Engl Ed. 2021;50(Suppl 1):64–72. 34281805 10.1016/j.rcpeng.2021.01.001 PMC8666103

[33] Chorwe-Sungani G, Mwagomba M, Chirwa E, Jere D, Chipps J. Acceptability and feasibility of a screening protocol for antenatal depression (SPADe) in Blantyre District, Malawi. BMC Psychiatry. 2022;22(1):544. 35953774 10.1186/s12888-022-04195-5 PMC9371629

[34] Pence BW, Stockton MA, Mphonda SM, Udedi M, Kulisewa K, Gaynes BN, et al. How faithfully do HIV clinicians administer the PHQ-9 depression screening tool in high-volume, low-resource clinics? Results from a depression treatment integration project in Malawi. Glob Ment Health Camb Engl. 2019;6:e21. 10.1017/gmh.2019.22 PMC6796321 31662876

[35] McCauley M, Brown A, Ofosu B, van den Broek N. I just wish it becomes part of routine care: healthcare providers’ knowledge, attitudes and perceptions of screening for maternal mental health during and after pregnancy: a qualitative study. BMC Psychiatry. 2019;19(1):279. 31500606 10.1186/s12888-019-2261-x PMC6734443

[36] Martin F, Nalukenge W, Lazarus O, Birungi J, Seeley J. Vital: HIV counselling and testing staff’s views of addressing mental health with HIV in Uganda. BMC Health Serv Res. 2020;20(1):1027. 33172447 10.1186/s12913-020-05881-4 PMC7654166

[37] Martin F, Clowes E, Nalukenge W, Clark C, Lazarus O, Birungi J, et al. Exploring the extent of mental health practice: content and cluster analysis of techniques used in HIV testing and counselling sessions in Uganda. AIDS Care. 2022;1–7. 10.1080/09540121.2022.2077909 35603881

[38] Tseng A, Foisy M, Hughes CA, Kelly D, Chan S, Dayneka N, et al. Role of the pharmacist in caring for patients with HIV/AIDS: clinical practice guidelines. Can J Hosp Pharm. 2012;65(2):125–45. 22529405 10.4212/cjhp.v65i2.1120 PMC3329905

[39] Organisation internationale de la francophonie. La langue française Dans Le monde: 2019–2022. Paris: Éditions Gallimard; 2022. editor.

[40] Zotova N, Watnick D, Awoh AR, Nsonde DM, Moungang EFT, Noumedem JLN, et al. Understanding the PHQ-9 items and depression among people living with HIV: A multiple methods study in Yaoundé, Cameroon. under review. 10.1016/j.ssmmh.2024.100353 PMC11694725 39749043

[41] Nasir LS, Al-Qutob R. Barriers to the diagnosis and treatment of depression in Jordan. A nationwide qualitative study. J Am Board Fam Pract. 2005;18(2):125–31. 15798141 10.3122/jabfm.18.2.125

[42] Dadi AF, Miller ER, Azale T, Mwanri L. We do not know how to screen and provide treatment: a qualitative study of barriers and enablers of implementing perinatal depression health services in Ethiopia. Int J Ment Health Syst. 2021;15:41. 33952338 10.1186/s13033-021-00466-y PMC8098000

