# Supplementary material for: Feasibility and acceptability of systematic screening for depression among people with HIV in Senegal: a qualitative study among various stakeholders
Source: BMC Psychiatry. 2026 Jan 27;26:180. doi: 10.1186/s12888-026-07812-9 (PMC12918477; doi:10.1186/s12888-026-07812-9)
Supplement: Supplementary file 1 — Supplementary Material 1 [file 12888_2026_7812_MOESM1_ESM.docx]

| 1. **INTERVENTION CHARACTERISTICS** | **Link with depression screening** | **Who is concerned** | | | | |
| --- | --- | --- | --- | --- | --- | --- |
|  |  | **PLHW** | **HPi** | **HPni** | **A** | **HS** |
| 1. Intervention Source | Studies have shown that the implementation of mental health interventions can be more or less accepted depending on who has implemented them: more accepted if it is a Ministry of Health rather than an external NGO, for example |  | × | × |  |  |
| 1. Evidence Strength & Quality | The fact that stakeholders are aware of and understand the issue/importance/effectiveness of screening for depression is a facilitator | × | × | × |  |  |
| 1. Relative Advantage | Not applicable here |  |  |  |  |  |
| 1. Adaptability | Can stakeholders adapt depression screening to their needs and contexts, or is the intervention too rigid to allow this?  What would need to be changed for the intervention to work effectively in this context? Are there elements of the intervention that should not be changed? (Interesting because we know that there is no systematic screening and that they carry it out in a targeted way) | × | × | × |  |  |
| 1. Triability | Not applicable here |  |  |  |  |  |
| 1. Complexity | Is depression screening complex to implement?  Are the people involved capable of dealing with it?  🡪 Questions about perceptions, difficulties encountered, etc.  🡪 Tool questions ++ | × | × | × |  |  |
| 1. Design Quality & Packaging | How has depression screening been presented to stakeholders?  What materials and tools are available to help with implementation? What is the stakeholders' perception of the quality of the materials and tools used to screen for depression ? How does this influence implementation? | × | × |  |  |  |
| 1. Cost | Not applicable here |  |  |  |  |  |

PLWH : People Living with HIV ; HPi : Healthcare professional involved in the project ; HPni : Healthcare professional non-involved in the project

A : Representative from PLWH associations ; HS : Representative from the healthcare system

| 1. **OUTER SETTING** | **Link with depression screening** | **Who is concerned** | | | | |
| --- | --- | --- | --- | --- | --- | --- |
|  |  | **PLHW** | **HPi** | **HPni** | **A** | **HS** |
| 1. Patient Needs & Resources | Are the needs of stakeholders (e.g. patients) known and prioritised? Does screening for depression meet these needs? | × | × | × | × | × |
| 1. Cosmopolitanism | How do formal and informal networks between health centres influence implementation? What kind of information, whether related to the intervention or more general? Do the players exchange information with others outside their health centre or with those who do not organise depression screening? |  | × | × |  |  |
| 1. Peer Pressure | Do health workers feel external pressure to implement screening for depression?  Question of implementation in other health centres, do they talk to each other within the centre or between centres? |  | × |  |  |  |
| 1. External Policy & Incentives | What policies, plans, programmes or regulations influence screening for depression?  Question of mental health guidelines (seen as a limitation in many studies: lack of clear guidelines) |  | × | × |  | × |

`

| 1. **INNER SETTING** | **Link with depression screening** | **Who is concerned** | | | | |
| --- | --- | --- | --- | --- | --- | --- |
|  |  | **PLHW** | **HPi** | **HPni** | **A** | **HS** |
| 1. Structural Characteristics | How does the size of the healthcare establishment and its conditions influence the implementation of depression screening?  🡪 Adequate space for screening | × | × | × |  |  |
| 1. Networks & Communications | How does information on depression screening circulate between the various stakeholders: the community, health workers, the district, the ministry, etc.?  How do relationships between stakeholders influence depression screening implementation?  🡪 Relationships between patients and health workers, between health workers and between patients regarding depression screening. | × | × | × | × |  |
| 1. Culture | How do different local cultures influence the implementation of depression screening?  🡪 Culture, stigma of depression | × | × | × | × |  |
| 1. Implementation Climate | How did the various local stakeholders initially react to the introduction of depression screening?  How did they welcome the change? How were the benefits of implementing this screening explained to them?  🡪 Perceptions of the relevance of depression screening | × | × | × |  |  |
| 1. Tension for Change | Is depression screening essential to meet the needs of patients and the objectives of HIV-care facilities? | × | × | × |  |  |
| 1. Compatibility | Is depression screening compatible with local values and the way in which health facilities work? How is screening for depression integrated into current operating processes?  🡪 Explaination of the stages of depression screening in real life and the feelings about supervision  🡪 Suggestions | × | × | × |  |  |
| 1. Relative Priority | Is depression screening more/less important than other interventions implemented at the same time?  🡪 Not necessarily to be explored here |  |  |  |  |  |
| 1. Organizational Incentives & Rewards | How do organisational rewards influence the implementation of depression screening? |  | × | × |  |  |
| 1. Goals and Feedback | Has the health centre set itself objectives in relation to depression screening? Does this influence its implementation? |  | × | × |  |  |
| 1. Learning Climate | Involvement of healthcare professionals for depression screening |  | × |  |  |  |
| 1. Readiness for Implementation | Stakeholders (health professionals) feel that they are essential/valued/competent to depression screening |  | × |  |  |  |
| 1. Leadership Engagement | Management commitment (supervision) and its impact on depression screening |  | × |  |  |  |
| 1. Available Resources | Questions about time to inform and train them for depression screening |  | × |  |  |  |
| 1. Access to Knowledge & Information | Access to knowledge and information (what do they do if they have questions about depression screening?) |  | × |  |  |  |

| **CHARACTERISTIC OF INDIVIDUALS** | **Link with depression screening** | **Who is concerned** | | | | |
| --- | --- | --- | --- | --- | --- | --- |
|  |  | **PLHW** | **HPi** | **HPni** | **A** | **HS** |
| 1. Knowledge & Beliefs about the Intervention | What do stakeholders know or do not know about depression screening? How do stakeholders perceive screening for depression? In their opinion, what are the advantages and disadvantages?  🡪 Question of important beliefs and perceptions ++  🡪 Frequently mentioned in literature | × | × | × | × | × |
| 1. Self-efficacy | Do health workers feel able/legitimate to screen for depression? |  | × | × |  |  |
| 1. Individual Stage of Change | Do health workers feel able/legitimate to screen for depression? |  | × |  |  |  |
| 1. Individual Identification with Organization | Do health workers support the intervention? Do people with more experience act differently from those with less? |  | × | × |  |  |
| 1. Other Personal Attributes | Not applicable here, could be considered in a second inductive approach here |  |  |  |  |  |

| **PROCESS** | **Link with depression screening** | **Who is concerned** | | | | |
| --- | --- | --- | --- | --- | --- | --- |
|  |  | **PLHW** | **HPi** | **HPni** | **A** | **HS** |
| 1. Planning | Were the activities related to depression screening planned in advance? What have they done to plan ahead? |  | × |  |  |  |
| 1. Engaging | Which stakeholders are involved in the implementation? What has been done to involve them? Who are they? |  | × |  |  |  |
| 1. Opinion Leaders | Qui sont les leaders d’opinion ? Comment influencent-ils la mise en œuvre ? A quel niveau se trouvent-ils ? Que disent-ils et comment font-ils pour influencer le dépistage de la dépression ? |  | × |  |  |  |
| 1. Formally Appointed Internal Implementation Leaders | Has a health worker been appointed as a "referent" in each centre to facilitate implementation? |  | × |  |  |  |
| 1. Champions | Not applicable |  |  |  |  |  |
| 1. External Change Agents | What external stakeholders influence the implementation of depression screening? |  | × |  |  |  |
| 1. Participants in the intervention | How do health workers inform patients about depression screening? Do patients know about it? | × | × | × |  |  |
| 1. Executing | How are the various activities being carried out (e.g. delivery of care, raising awareness in the community, keeping a diary/tool, etc.)?  Is it going as planned? Are the deadlines being met? | × | × |  |  |  |
| 1. Reflecting & Evaluating | What ongoing evaluations are planned and how are they carried out? What feedback is given to agents and how is this done? |  | × |  |  |  |
