# Supplementary material for: Feasibility and acceptability of systematic screening for depression among people with HIV in Senegal: a qualitative study among various stakeholders
Source: BMC Psychiatry. 2026 Jan 27;26:180. doi: 10.1186/s12888-026-07812-9 (PMC12918477; doi:10.1186/s12888-026-07812-9)
Supplement: Supplementary file 2 — Supplementary Material 2 [file 12888_2026_7812_MOESM2_ESM.docx]

###### Interview guide - Healthcare professional involved in the “Intervention Depression” project

**Introduction and presentation:**

Hello, my name is XX, and I'm a Masters student in public health in Bordeaux, France. I'm doing an internship in the research team working on the "Intervention depression" project in which you're involved.

I'd like to talk to you about the depression screening you've carried out among your patients living with HIV. We'd like to better understand the conditions under which this screening is carried out, so that as many patients as possible can benefit from it and receive appropriate care.

This interview will last about 30 minutes. If it suits you, the interview will be recorded so that I can properly transcribe what you have told me for my research.

I remind you that all this will remain confidential: you will be completely anonymous and your name will not appear anywhere.

You are free to end the interview whenever you wish, to let me know if a question bothers you, and you are free not to answer certain questions. There are no right or wrong answers, the aim here is to give you the floor to better understand the situation, without judgment.

Do you have a question or something you'd like to tell me before we start?

1. **Socio-demographic characteristics**

- First of all, can you describe who you are?
  - Gender
  - Age
  - Profession
  - Number of years' experience in this trade
  - Years of experience working with PLWHA

1. **Professional and mental health experience**

- Can you describe your experience as a mediator/doctor/social worker?
- Can you tell me about your career path, from the end of your studies to your arrival at the center?
- Can you tell us about your experience in mental health?
  - On a personal level
  - On a professional level
  - At community or association level

1. **Perception of the project and depression**

- Can you tell me about the interpersonal group therapy project?
  - Opinion on the project
  - Reaction to the project announcement
  - Understanding the project
  - Perception of mental health, depression

1. **Depression management**

- Can you describe for me the circuit for managing depression in PLWH patients in general? And then refocus (before/after, project or non-project, within the structure, at national level and according to the type of patient).
- How are you involved in this process?
  - Involvement
- Can you tell me about the screening process for depression?
- What do you think of the depression screening stage?
  - General feeling about this stage and its usefulness
  - Feelings about the tool used (simplicity, length of test, understanding of items, possible difficulties, etc.)
- What do you think are the repercussions of this screening?
  - Benefit (for patients, for them)
  - Usefulness
  - Understanding the issue
  - Load (work, time)
  - Coherent with the center, adapted to the needs and population

1. **Determinants of implementation**

- What successes or difficulties have you encountered when screening for depression?
  - Tool
  - Keeping the register
  - Refusal
  - Workload
  - Lack of time
  - Lack of space
  - Culture and stigma
  - Knowledge
  - Legitimacy (self-efficacy)
- If you've encountered difficulties, how have you dealt with them?
  - Answers, adaptation
  - Change in context
- Have you been able to talk about your experience of carrying out this screening? (With colleagues, supervisors, peers in other centers)
  - Supervision, management

1. **Areas for improvement**

- What do you think could be done to improve depression screening?
- What should/should not be changed (treatment circuit, location, type of personnel, etc.)?
- In your opinion, how could this screening be implemented on a long-term basis (outside the project)? And systematically and/or regularly during follow-up (integrated into the service)?
  - Suggestions

1. **Conclusion of the interview**

- Do you have anything to add? Anything you'd like to talk about? Your opinion of the interview? Something you'd like to come back to?

**Thanks**

###### Interview guide - Healthcare professional who did not take part in the project

Circumstances of the interview :

**Introduction and presentation:**

Hello, my name is XX, and I'm a Masters student in public health in Bordeaux, France. I'm doing an internship in the research team working on the "Intervention depression" project, which is being set up in the center where you work.

I'd like to talk to you about screening for depression in people living with HIV, to see if and how we can make this available to as many patients as possible so that they can be managed appropriately.

This interview will last about 30 minutes. If it suits you, the interview will be recorded so that I can properly transcribe what you have told me for my research. I remind you that all this will remain confidential: you will be completely anonymous and your name will not appear anywhere.

You are free to end the interview whenever you wish, to let me know if a question bothers you, and you are free not to answer certain questions. There are no right or wrong answers, the aim here is to give you the floor to better understand the situation, without judgment.

Do you have a question or something you'd like to tell me before we start?

1. **Socio-demographic characteristics**

- First of all, can you describe who you are?
  - Gender
  - Age
  - Profession
  - Number of years' experience in this trade
  - Years of experience working with PLWHA

1. **Professional and mental health experience**

- Can you describe your experience as a mediator/doctor/social worker?
- Can you tell me about your career path, from the end of your studies to your arrival at the center?
- Can you tell us about your experience in mental health? (Personal, professional)
  - Knowledge and beliefs about mental health

1. **Perception of the project and depression**

- Have you heard about the interpersonal group therapy project? Can you tell me about it?
  - Understanding the project
  - Perception of mental health, depression
  - Knowledge of depression
  - Perceived relevance of this management (compared with management of other comorbidities)
  - Benefits and consequences for patients and healthcare professionals

1. **Perceptions of depression management**

- Can you describe for me the circuit for managing depression in PLWHA patients in general? And then refocus (before/after, project or non-project, within the structure, at national level and according to the type of patient).
- How are you involved in this process?
  - Involvement
- What do you think is important to consider when managing depression?
  - Obstacles
  - Facilitators
- Can you tell me what the depression screening stage means to you?
  - Perceived feasibility and acceptability to patients and healthcare professionals
- Would you feel legitimate to carry out this screening? (Some pressure, question of training and capabilities, self-efficacy, workload, type of professional to carry out this screening, scaling up)

1. **Recommendations**

- In your opinion, how could this screening be systematically implemented in the center?
  - Suggestions (care circuit, location, type of personnel, etc.)

1. **Conclusion of the interview**

- Do you have anything to add? Anything you'd like to talk about? Your opinion of the interview? Something you'd like to come back to?

**Thanks**

Circumstances of the interview :

###### Interview guide - PLWHA association representatives

**Introduction and presentation :**

Hello, my name is XX, and I'm a Masters student in public health in Bordeaux, France. I'm doing an internship with the research team working on the "Intervention depression" project, which is being implemented in 4 centers in and around Dakar. This is a project to treat depression in people living with HIV. Patients can benefit from group therapy if they are eligible, i.e. if they show depressive symptoms when screened for depression.

I'd like to talk to you about the assessment and management of depressive symptoms in people living with HIV, with a view to improving their care.

This interview will last about 30 minutes. If it suits you, the interview will be recorded so that I can properly transcribe what you have told me for my research. I remind you that all this will remain confidential: you will be completely anonymous and your name will not appear anywhere.

You are free to end the interview whenever you wish, to let me know if a question bothers you, and you are free not to answer certain questions. There are no right or wrong answers, the aim here is to give you the floor to better understand the situation, without judgment.

Do you have a question or something you'd like to tell me before we start?

1. **Socio-demographic characteristics**

- First of all, can you describe who you are?
  - Gender
  - Age
  - Profession
  - Education level
  - Marital status
  - Religion
  - Ethnic group

1. **Professional and associative experience**

- Could you describe your career path, from the end of your studies to your arrival at the association?
- Can you describe the association you are involved with?
- Can you describe your current role within the association?

1. **Experience and perception of mental health**

- Can you tell us about your experience in mental health? (Personal, professional)
- What does mental health mean to you?
- What does depression mean to you?
  - Knowledge and beliefs about mental health and depression
- What can you tell me about the mental health of PHAs?
  - Mental health and HIV link

1. **Mental health in the association**

- What role does mental health play in your association?
  - Perception of the issue
  - The question of priority
- How do we talk about it?
- What actions are you taking or planning to take?
- Have the association's patients ever expressed mental health needs to you? How did they formulate these needs? What have you done to respond?
- Do patients talk to each other about mental health/depression?
- Do patients know how to manage depression?

1. **Perception of depression screening**

- How do you feel about screening for depression in PHAs?
  - Perception of usefulness
- In your opinion, who could perform this screening, at what point(s) in the care of PLWHA and where?
- In your opinion, what problems might be encountered in setting up systematic screening for depression among PLWHA?
- What could make it easier to set up?
  - Obstacles
  - Facilitators
  - Suggestions

1. **Conclusion of the interview**

- Do you have anything to add? Anything you'd like to talk about? Your opinion of the interview? Something you'd like to come back to?

**Thanks**

Circumstances of the interview :

###### *Interview guide - Health system representatives*

**Introduction and presentation:**

Hello, my name is XX, and I'm a Masters student in public health in Bordeaux, France. I'm doing an internship with the research team working on the "Intervention dépression" project, which is being implemented in 4 centers in and around Dakar. This is a project to treat depression in people living with HIV. Patients can benefit from group therapy if they are eligible, i.e. if they show depressive symptoms when screened for depression.

I'd like to talk to you about depression screening for people living with HIV in order to improve their care.

This interview will last about 30 minutes. If it suits you, the interview will be recorded so that I can properly transcribe what you have told me for my research. I remind you that all this will remain confidential: you will be completely anonymous and your name will not appear anywhere.

You are free to end the interview whenever you wish, to let me know if a question bothers you, and you are free not to answer certain questions. There are no right or wrong answers, the aim here is to give you the floor to better understand the situation, without judgment.

Do you have a question or something you'd like to tell me before we start?

1. **Socio-demographic characteristics**

- First of all, can you describe who you are?
  - Age
  - Profession

1. **Professional experience**

- Could you describe your career path, from the end of your studies to your current position?
- Can you describe your current position?
- Can you describe your role in managing this program (PNLS, DSM)?
  - Tasks to be performed
  - What they see, what they have to deal with

1. **Mental health and HIV**

- How does mental health fit into this program?
  - Knowledge
  - Perception of the issue
- What do you think is the link between mental health and HIV?
  - Perception of mental health and depression among people living with HIV
  - Beliefs and knowledge

1. **Depression management and screening**

- What do you think about mental health care for people living with HIV? Depression in particular?
  - Priority (over other comorbidities)
  - Importance
  - Implementation (feasibility)
- How do you feel about screening for depression in PHAs?
  - Perception of usefulness
- In your opinion, who could perform this screening, at what point(s) in the care of PLWHA and where?
- In your opinion, what problems might be encountered in setting up systematic screening for depression among PLWHA?
- What could make it easier to set up?
  - Issues
  - Implementation challenge
  - Difficulties
  - Workload
  - Lack of resources
  - Suggestions

1. **Conclusion of the interview**

- Do you have anything to add? Anything you'd like to talk about? Your opinion of the interview? Anything you'd like to come back to?

**Thanks**

Circumstances of the interview :

###### Interview guides - PLWH who have received or are about to receive group IPT

**Introduction and presentation:**

Hello, my name is XX, and I'm a Masters student in public health in Bordeaux, France. I'm doing an internship in the research team working on the "Intervention depression" project in which you're taking part.

[I'd like to talk to you about your experience with this project, and in particular about the time when the [health teacher] assessed your depressive symptoms to see if you could take part in the project. Indeed, we would like to evaluate how this type of assessment should be implemented on a more regular basis during your follow-up.

This interview will last about 30 minutes. If it suits you, the interview will be recorded so that I can properly transcribe what you have told me for my research. I remind you that all this will remain confidential: you will be completely anonymous and your name will not appear anywhere.

You are free to end the interview whenever you wish, to let me know if a question bothers you, and you are free not to answer certain questions. There are no right or wrong answers, the aim here is to give you the floor to better understand the situation, without judgment.

Before proceeding, I suggest you fill in a consent form to give me your written agreement.

Do you have a question or something you'd like to tell me before we start?

1. **Socio-demographic characteristics**

- First of all, can you describe who you are?
  - Gender
  - Age
  - Profession
  - Education level
  - Marital status
  - Religion
  - Ethnic group
  - Place of residence

1. **Therapy, perception and understanding of depression**

- How long have you been in therapy? (Or how long have you known you were going to be in therapy, if you haven't started yet).
- Can you tell me why you joined this therapy group?
  - Understanding why they are in a therapy group
  - Awareness of depression
  - Knowledge of depression (before therapy vs now)
  - Perception of depression, what it evokes in them (question of whether they knew anyone, how others reacted, their care)
  - Link between depression and HIV infection

1. **Perceptions of depression screening**

- How did the assessment of your depressive symptoms go?
  - Steps: PHQ-9, doctor's appointment
  - Who performed the test, if appropriate (relationship with healthcare professional)
  - Approach/attitude of the healthcare professional
  - Explanation of what it consists of and its purpose
  - Perceptions of PHQ-9: understanding questions, reformulations
- How did you feel then?
  - Experience of the moment (screening and diagnosis), feelings when answering questions
  - In relation to the time
  - In relation to location
  - Explore feelings (fear, apprehension, incomprehension, shame...)
  - Stigmatization (by family and friends, healthcare professionals, other patients, etc.)
- In your opinion, what is the purpose of this screening/its place/its impact on your care?
  - Perceived consistency of intervention with needs (and medical priorities)
  - Perception of the importance of screening
  - Understanding that this enables access to mental health care
  - Perceived usefulness

1. **Suggestions for improving screening for depression**

- What do you think could be done to improve depression screening?
- What should/should not be changed (treatment circuit, location, type of personnel, etc.)?
- What approach would you have preferred? How would you have liked it?
  - Suggestions at various levels (on the health personnel who carry out the screening, how to do it, where, when...)

1. **Conclusion of the interview**

- Do you have anything to add? Anything you'd like to talk about? Your opinion of the interview? Something you'd like to come back to?

**Thanks**

Circumstances of the interview :
