# Supplementary material for: Feasibility and acceptability of systematic screening for depression among people with HIV in Senegal: a qualitative study among various stakeholders
Source: BMC Psychiatry. 2026 Jan 27;26:180. doi: 10.1186/s12888-026-07812-9 (PMC12918477; doi:10.1186/s12888-026-07812-9)
Supplement: Supplementary file 3 — Supplementary Material 3 [file 12888_2026_7812_MOESM3_ESM.docx]

###### Coding tree

| **0. Coding according to objectives** | Barriers | |
| --- | --- | --- |
|  | Facilitators | |
|  | Requirements | |
| **1. Intervention features** |  | |
|  | 1.1 Origin of the intervention | - Perceptions of the origin of the intervention   - Positive - Negative |
|  | 1.2 Quality and strength of evidence | - Perceptions of the importance of depression screening  -Understanding of the issue and opinion on effectiveness  - Opinion on the relevance of developing this intervention in the service, in general  - Perceptions of intervention in relation to existing scientific data |
|  | 1.4. Adaptability | - Use of depression screening, coping strategies employed |
|  | 1.5. Complexity | - Perception and viewpoint of the complexity of implementing systematic screening for depression, difficulties encountered  - Perception of the tool used |
|  | 1.6. Quality and presentation of the intervention model | - Perception of how the intervention was set up and presented in the department |
| **2. External context** | 2.1. Patient needs and resources | - Patients' perceived need for depression screening, and more generally for mental health care. |
|  | 2.2. Network | - Exchanges between different centers/actors on the subject of depression screening |
|  | 2.3. Peer pressure | - External/internal pressure to implement depression screening |
|  | 2.4 External incentives or policies | - Perceptions of mental health plans, programs and policies  - Allocated resources |
|  | Global context: system | - Beliefs, religions |
| **3. Internal context** | 3.1 Structural features | - Size and structure of health center influencing implementation |
|  | 3.2. Networks and communications | - How information on depression screening is circulated between stakeholders (community, healthcare professionals, patients)  - Patient-healthcare professional relations |
|  | 3.3 Culture | - Influence of local culture on the implementation of depression screening :   - Stigma related to HIV, mental health - Community perception of mental health and depression - The role of traditional practitioners |
|  | 3.4. Location climate | - Stakeholders' reactions to the introduction of systematic depression screening: how did they react to the change?  - How depression screening is integrated, or can be integrated (compatibility with values, ways of doing things, in current operating processes):   - Which practitioner - Operation within the structure: stages - At what point   - Relative priority (depression vs. other comorbidities)  - Organizational incentives and rewards |
|  | 3.5. Preparing for installation | - Executive commitment  - Resources available for implementation :   - Time - Training - Physical Space - Education |
| **4. Individual characteristics** | 4.1. Knowledge and beliefs about intervention | - Knowledge and beliefs about depression, mental health and screening :   - Patients - Healthcare professionals - Association - Health system representative |
|  | 4.2. Self-efficacy | - Individuals' belief in their own ability to perform depression screening |
|  | 4.3 Individual stage of change | - Adherence to intervention, motivation |
|  | 4.4 Individual identification with the organization | - Involvement in the organization's activities and its influence on implementation |
| **5. Process** | 5.1. Planning | - Plans, methods, pre-planned tasks |
|  | 5.2 Involvement | - Involvement of opinion leaders, internal leaders  - Involvement of participants  - Involvement of external leaders (associations) |
|  | 5.3. Execution | - Screening for depression  - How to proceed |
|  | 5.4. Reflection and evaluation | - Intervention evaluation  - Feedback |

### 
